# “Quiescence” in the resting zone of the growth plate: a systematic review

**DOI:** 10.1093/stmcls/sxag010

**Published:** 2026-03-07

**Authors:** Mahtab Avijgan, Amal Nazaraliyev, Klas Blomgren, David Gomez-Cabrero, Phillip T Newton

**Affiliations:** Department of Women’s and Children’s Health, Karolinska Institutet, 171 77 Stockholm, Sweden; Astrid Lindgren Children’s Hospital, Karolinska University Hospital, Region Stockholm, 171 64 Stockholm, Sweden; Department of Women’s and Children’s Health, Karolinska Institutet, 171 77 Stockholm, Sweden; Astrid Lindgren Children’s Hospital, Karolinska University Hospital, Region Stockholm, 171 64 Stockholm, Sweden; Department of Women’s and Children’s Health, Karolinska Institutet, 171 77 Stockholm, Sweden; Pediatric Oncology, Karolinska University Hospital, 171 76 Stockholm, Sweden; Division of Biomedical Sciences, King Abdullah University of Science and Technology (KAUST), Thuwal, 23955-6900, Saudi Arabia; Department of Women’s and Children’s Health, Karolinska Institutet, 171 77 Stockholm, Sweden; Astrid Lindgren Children’s Hospital, Karolinska University Hospital, Region Stockholm, 171 64 Stockholm, Sweden

**Keywords:** cellular quiescence, growth plate, resting zone, skeletal stem cells, chondrocyte

## Abstract

Postnatal skeletal growth in childhood and adolescence depends on cartilage organs called (epiphyseal) growth plates. Studies in the last decade have identified populations of skeletal stem cells within mouse growth plates′ resting zones. While cellular quiescence is vital for the maintenance of many tissue-resident stem cell populations, the resting zone chondrocytes have been labeled “quiescent” for decades. However, the features of cellular quiescence that have been reported in the postnatal resting zone, how they were defined or experimentally assessed, and knowledge gaps relative to other quiescent cell types, remain to be well described. To address this, we conducted a systematic review, using the PRISMA guidelines, to identify studies of resting zone chondrocytes including the prefix “quiescen*.” Definitions, keywords, chronological data and experimental findings were extracted. Our analysis demonstrated that, compared to those in other well-studied tissues, features of cellular quiescence in RZ chondrocytes remain poorly reported and underexplored, with limited molecular and functional characterization. Furthermore, while most identified studies reported changes in cell division parameters, integration between cues controlling resting zone cell quiescence is incomplete and heterogeneity among the various sub-populations of RZ cells/potential quiescent states is yet to be fully determined. This review identifies consensuses and knowledge gaps between studies and between quiescent RZ cells and those in other tissues and can act to enhance consistency and comparability in future studies of “quiescence” in the RZ chondrocytes.

## Graphical Abstract

**Graphical Abstract:**
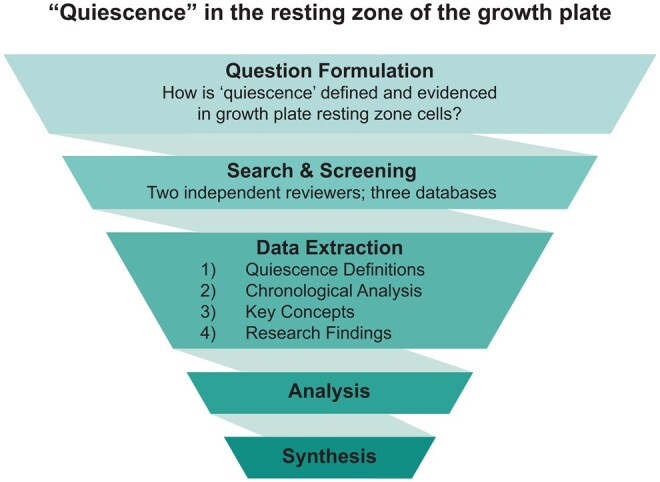

Significance statement Bone elongation occurs via cartilage organs called (epiphyseal) growth plates. Resting zones within growth plates have long been known to contain “quiescent” chondrocytes, but only in recent years have populations of skeletal stem cells been identified within them. We conducted a systematic review to identify historically described features of cellular quiescence and contextualized them with current insights from the growth plate and cellular quiescence fields. This enabled us to catalog *bona fide* quiescent features of resting zone cells and highlight open questions, including undiscovered indicators, heterogeneity, and regulatory mechanisms—issues with broad relevance to pediatrics and stem cell biology.

## Introduction

Bone elongation during childhood and adolescence is caused by narrow cartilage organs called growth plates, which form bones via a cartilage intermediate in the process of endochondral ossification^1^ (Note Supplementary Information). The resting zone (RZ) of the growth plate is crucial for post-natal skeletal elongation as it houses a unique population of skeletal stem cells that drives bone elongation. While the identification of *bona fide* stem cells and their niche occurred in recent years,^2–4^ the term “quiescence” has frequently been used as an adjective to describe RZ cells for many decades. “Quiescence” is rooted in the Latin word “quiescentia,” stemming from the verb “quiescere,” which means “to rest” or “to be quiet.”^5^ The word “quiescent” was first applied to cells of low proliferation rates by Clowes in the mid-1950s.^6^ Hence, when referred to, in relation to cell biology, “quiescence” has come to mean cells with relatively low rates of cell division, which maintain the potential to later proliferate, also more precisely described as “cellular quiescence.”^7^

While reversible cell cycle arrest remains the basis of the term “cellular quiescence,” it has developed further meaning over time as additional features common to quiescent cells across different tissues have been discovered. Research since the 1950s has revealed cellular quiescence to be a dynamic, actively maintained state with diverse features. Quiescent cells, such as quiescent skeletal muscle stem cells and adult neural stem cells, are smaller than their active counterparts, with reduced RNA/protein content and suppressed mRNA transcription, reflecting their low biosynthetic activity.^8–11^ They exhibit low levels of cyclins (A2, B1, E2) and high levels of CDK inhibitors (p21, p27, p57), which enforce cell cycle arrest by blocking cyclin-CDK activity.^12–14^ Quiescent muscle, neural, and hair follicle stem cells (MuSCs, NSCs, HFSCs) display dense heterochromatin visible via electron microscopy, indicating transcriptionally repressive chromatin states.^15–17^ Many genes in quiescent cells harbor both repressive (H3K27me3) and permissive (H3K4me3) histone modifications, keeping them “poised” for rapid activation during re-entry into the cell cycle.^18–22^ Quiescent HSCs, MuSCs, NSCs, and HFSCs have low mitochondrial activity^23–26^ and reduced oxidative metabolism^27–29^ due to mitophagy (mitochondrial autophagy), aligning with their minimal energy demands.^30^^,^^31^ Residing in low-oxygen microenvironments, these cells rely primarily on glycolysis rather than oxidative phosphorylation to meet energy needs.^32–36^ Quiescent cells often show enlarged lysosomes and upregulated expression of lysosomal genes, suggesting increased lysosomal activity compared to proliferative states.^27^^,^^37–40^ The proposed roles of lysosomes in quiescence include degrading growth-factor receptors to maintain quiescence or mitigating stress via ROS reduction, mitochondrial degradation, and toxin removal.^27^^,^^37^^,^^38^^,^^40^ Conversely, lysosomes may become sources of carbon mass during quiescence, releasing it during cell-cycle re-entry to fuel proliferation, highlighting their dual potential roles.^27^^,^^40^ Altogether, these studies show the depth of understanding relating to features of cellular quiescence across various tissues and species, and its vital role in maintaining the balance of stem cell populations.

Emerging evidence supports the existence of quiescence subtypes in vivo. Neural stem cells exhibit a “Neural G0” state characterized by unique transcriptional profiles and functional properties distinct from other non-dividing states.^41^ Cancer models in melanoma and glioblastoma reveal subpopulations of quiescent cells with divergent gene expression patterns and varying depths of cell cycle arrest.^42^^,^^43^ This heterogeneity adds complexity to the study of cellular quiescence, as different subtypes may respond uniquely to reactivation signals or therapeutic interventions. The notion that quiescence is not a single state but a continuum^44^—meaning that the same cell can exist in a range of quiescence states at different times, with varying molecular characteristics^42^^,^^43^—adds greater complexity to population identity and maintenance.

Hence, in the decades since RZ cells started to be labeled “quiescent,” based on their relatively low rates of cell division^45–47^ both the identity of RZ chondrocytes as stem cells and the concept of cellular quiescence have hugely transformed. Here, we conducted a systematic review to unearth the “quiescent” features of RZ cells reported throughout the years to assess their current and future relevance to the field.

## Methods

For this review, we implemented the Preferred Reporting Items for Systematic Reviews and Meta-Analyses (PRISMA) guidelines.^48^ The main objective of this review is to answer the question: How is ‘quiescence’ defined and evidenced in growth plate resting zone cells? The secondary objectives to be answered by this investigation include: (1) in studies of growth plate RZ chondrocytes, what features of cellular quiescence have been reported using the prefix “quiescen”? (2) How have these features been defined or experimentally investigated? (3) What knowledge gaps exist relative to quiescence in other stem cell populations? This review was not pre-registered in a public database during preparation.^48^

### Information sources

On October 27, 2024, we conducted a comprehensive literature search using PubMed, Embase, and Web of Science. The publication date was left at its default setting to maximize search coverage, enabling us to capture the full spectrum of available literature—from historical studies to the most recent publications. The literature search was updated on May 27, 2025.

### Search strategy

**Figure 1.**
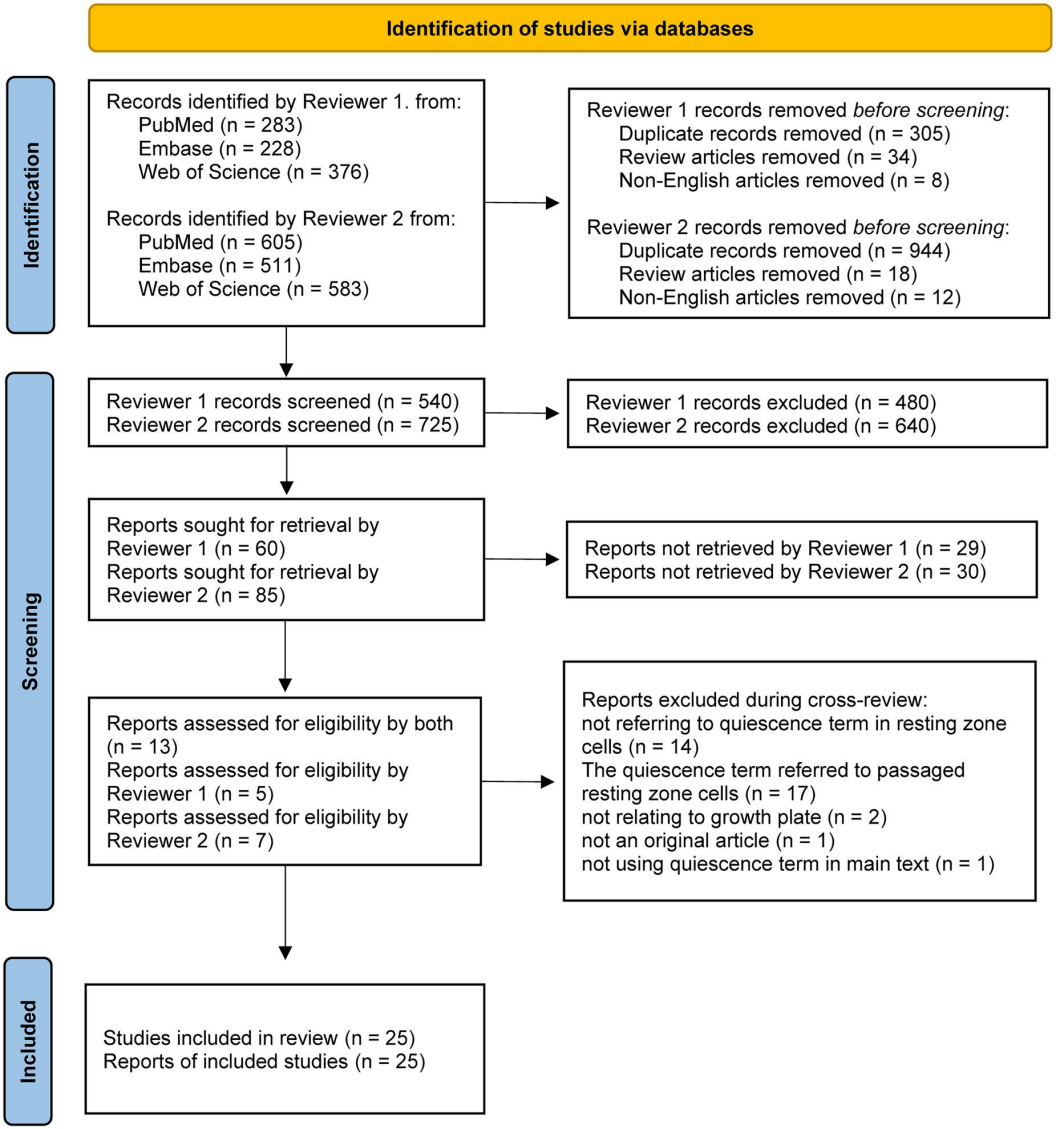
PRISMA flow diagram used to identify studies.

Literature searches were performed independently by two reviewers; M.A. (reviewer 1) and P.T.N. (reviewer 2), who entered free text or standardized keywords, which were used for the systematic search (Table S1) with the intention to identify all publications in which the term “quiescence” or “quiescent” was used in relation to resting zone chondrocytes. A validation set was devised by selecting four studies that reviewer 1 knew mentioned “quiescence” or “quiescent” and studied the RZ of the growth plate.^49–52^ The search strategy was validated by checking that these four eligible studies in the validation set had been identified by both reviewers in their independent searches. The language was limited to English and review articles were excluded from the search. Duplicates and publications that were neither open access nor available through Karolinska Institutet’s library were excluded (Figure 1).

Data items for search validation: A validation set of four known eligible studies^49–52^ confirmed search strategy sensitivity, each was independently identified by both reviewers. The prefix “quiescen*” search terms specifically targeted RZ chondrocyte literature (Table S1).

### Eligibility criteria, selection process, and data synthesis

Research publications were selected based on pre-defined eligibility criteria (Figure 1). This can be summarized by inclusion: (1) the prefix “quiescen” was used in the main text, (2) the prefix “quiescen” was used in relation to RZ chondrocytes, and exclusion: (1) review articles, (2) non-English publications, (3) duplicates, (4) publications not accessible via open access or Karolinska Institutet library, (5) studies using passaged primary chondrocytes or cell lines (to prioritize in vivo relevance) (Note S2).

The reviewers independently screened the records by full text for suitability according to the eligibility criteria. The list of each reviewer’s included studies was blinded by mixing included with excluded studies and given to the other reviewer to cross-review for inclusion. There were no discrepancies between the reviewers′ opinions on inclusion regarding individual publications. Mutually included studies were subsequently pooled for data collection.

Data extraction and synthesis were performed as follows:

1. Quiescence definitions: The prefix “quiescen” was searched in the main text of all included studies, and full sentences with the term were recorded in a Word document to analyze the context in which the term was used (Table S2).
2. Chronological analysis: The year of publication was recorded for all included studies to identify any evolution in the use of term quiescence in the RZ of growth plate. Bar charts in Figure S2 were plotted using Excel (Version 2505 Build 16.0.18827.20102)
3. Key themes and concepts: All 25 included studies were eligible for synthesis. Only Embase and Web of Science databases allowed the exporting of keywords for our included studies. Embase and Web of Science keywords were obtained by exporting records of “Author keywords” into an Excel document. To visualize the key concept(s) across the studies, we used the extracted Keywords to generate a word cloud figure using RStudio version 4.1.3 (2022-03-10).
4. Research findings: Only studies that included experimental investigation of cellular quiescence in the RZ of the growth plate were eligible for synthesis. Steps to minimize bias are described in Note S3.

## Results

### Study selection

In the Identification step, all the records identified by the Reviewers were pooled, and duplicates, reviews and non-English articles were removed, prior to Screening. The difference in the number of articles obtained by each reviewer is due to their choice of search terms and strategy (Table S1). Upon collation, 35 identified studies were excluded as detailed in Table S3.

### Study characteristics

Among the 25 studies included in our systematic review, 13 mentioned “quiescence” or “quiescent” without making it a central focus of their research, while this concept was the research focus of the twelve remaining studies. A summary of the data items collected from these studies is presented in Table 1.

**Table 1.** Individual study analysis on quiescence definition.

No.	Study	Title of study	Is “quiescence” or “quiescent” defined explicitly?	Is a reference used to define “quiescence” or “quiescent”? If so, add the reference(s).	Is “quiescence” or “quiescent” accompanied by other definitions?	Was cellular quiescence investigated in the RZ?
**1**	53	Shared role of the pRB-related p130 and p107 proteins in limb development	No	Yes^54^	No	Yes
**2**	55	Identification and characterization of various differentiative growth plate chondrocytes from porcine by countercurrent centrifugal elutriation	No	No	Yes, “resting chondrocytes were found to be small in size and quiescent”; “homogeneous in appearance and represent the quiescent form”	No
**3**	56	Vascular endothelial growth factor (VEGF) in cartilage neovascularization and chondrocyte differentiation: auto-paracrine role during endochondral bone formation	No	No	No	No
**4**	57	Expression of galanin and galanin receptor-1 in normal bone and during fracture repair in the rat	No	Yes	Yes, “largely quiescent and only sporadically divide”	No
**5**	58	A thyrotoxic skeletal phenotype of advanced bone formation in mice with resistance to thyroid hormone	No	Yes^59^	No	No
**6**	60	Fibroblast growth factor expression in the postnatal growth plate	No	No	No	No
**7**	52	Gradients in bone morphogenetic protein-related gene expression across the growth plate	No	No	No	No
**8**	61	Sequential and Coordinated Actions of c-Myc and N-Myc Control Appendicular Skeletal Development	No	No	Yes, “largely quiescent chondrocytes”	No
**9**	49	G-protein stimulatory subunit alpha and Gq/11α G-proteins are both required to maintain quiescent stem-like chondrocytes	No	No	No	Yes
**10**	62	Loss of VHL in mesenchymal progenitors of the limb bud alters multiple steps of endochondral bone development	No	No	No	No
**11**	63	Multi-scale finite element model of growth plate damage during the development of slipped capital femoral epiphysis	No	No	Yes, “in a quiescent state and are smaller than those of the other zones”	No
**12**	64	Downregulation of basic fibroblast growth factor is associated with femoral head necrosis in broilers	No	No	Yes, “a highly dynamic sequence of quiescent stem cells/primordial chondrocytes (resting zone)”	No
**13**	3	Chondrocytes in the resting zone of the growth plate are maintained in a Wnt-inhibitory environment	No	Yes^49^	Yes “quiescent stem-like chondrocytes”	Yes
**14**	65	Depth and strain rate-dependent mechanical response of chondrocytes in reserve zone cartilage subjected to compressive loading	No	No	No	No
**15**	50	A FoxA2+ long-term stem cell population is necessary for growth plate cartilage regeneration after injury	No	Yes^66^	Yes, “a more quiescent, LTSSC population” (long-term skeletal stem cells: LTSSC); also contextualized with references to studies demonstrating slow-cycling nature of RZ cells in animal models.	Yes
**16**	67	Loss-of-function variant in SPIN4 causes an X-linked overgrowth syndrome	No	No	No	Yes
**17**	68	Investigating the interfaces of the epiphyseal plate: An integrated approach of histochemistry, microtomography, and scanning electron microscopy (SEM)	No	No	Yes, “a relatively quiescent reserve zone”	No
**18**	69	Hedgehog activation promotes osteogenic fates of growth plate resting zone chondrocytes through transient clonal competency	No	No	No	Yes
**19**	70	The G protein-coupled receptor ADGRG6 maintains mouse growth plate homeostasis through IHH signaling	No	No	No	Yes
**20**	51	Gli1 labels progenitors during chondrogenesis in postnatal mice	No	Yes^49^^,^^66^^,^^71^^,^^72^	Yes, “quiescent stem-like chondrocytes”; “As a quiescent or slowly proliferative cell population”	Yes
**21**	73	PI15, a novel secreted WNT-signaling antagonist, regulates chondrocyte differentiation	No	Yes^2–4^^,^^74^	Yes, “quiescent skeletal stem cells slowly cycle”	No
**22**	75	Ptip safeguards the epigenetic control of skeletal stem cell quiescence and potency in skeletogenesis	No	Yes^2^^,^^18^^,^^76–79^	Yes, “quiescence and potency”	Yes
**23**	80	Apolipoprotein E is a marker of all chondrocytes in the growth plate resting zone	No	Yes^81^	Yes, “Ccnd1 labels chondrocytes entering the cell cycle from the quiescent state”; “resting” chondrocytes are quiescent with a longer cell cycle	Yes
**24**	82	An epitranscriptomic program maintains skeletal stem cell quiescence via a METTL3-FEM1B-GLI1 axis	No	No	Yes, “quiescence and potency”; “quiescence and multipotency.”	Yes
**25**	83	Timing of resting zone parathyroid hormone-related protein expression affects maintenance of the growth plate during secondary ossification: a computational study	No	No	Yes “quiescent cells above the proliferative zone”; “a region of quiescent cells below the SOC is defined as the resting zone”	Yes

### Results of synthesis

#### Quiescence definitions

We found that none of the 25 included studies defined “quiescence” and less than half of the articles provided a context for its use (Table 1), either by referencing published articles or other descriptions. While we relied on direct quotations (Table S2) and had a small sample size of studies that provided implicit definitions (Table 1, *n* = 14), features associated with the “quiescent” RZ cells related to either (1) their low rates of cell division, (2) their small size, and (3) their stemness.

#### Chronological analysis

We were interested to understand if and how the use of the term “quiescence” in relation to the RZ of the growth plate had changed over time (Figure S2A), particularly in reference to the identification of *bona fide* stem cells and a stem cell niche in the postnatal epiphyseal growth plate in the late 2010s.^2^^,^^4^^,^^84^^,^^85^ To visualize this, we plotted the studies relating to the three features determined in “Quiescence definitions” section: (i) low rates of cell division,^3^^,^^50^^,^^51^^,^^57^^,^^61^^,^^68^^,^^73^^,^^80^^,^^83^ (ii) small cell size,^55^^,^^63^ (iii) stemness/differentiation potential^64^^,^^75^^,^^82^ by publication year (Figure S2B). While the number of observations is too few to draw meaningful conclusions about shifting understanding over time, even in these most recent studies, quiescence in relation to the RZ is still primarily used to mean a reversible, non-proliferative or slowly proliferative state without extensive characterization of its broader features beyond cell size. While cell size has not been used to describe RZ cells since 2015, features relating to stemness have only been mentioned as a feature since this time.

#### Research themes and concepts

**Figure 2.**
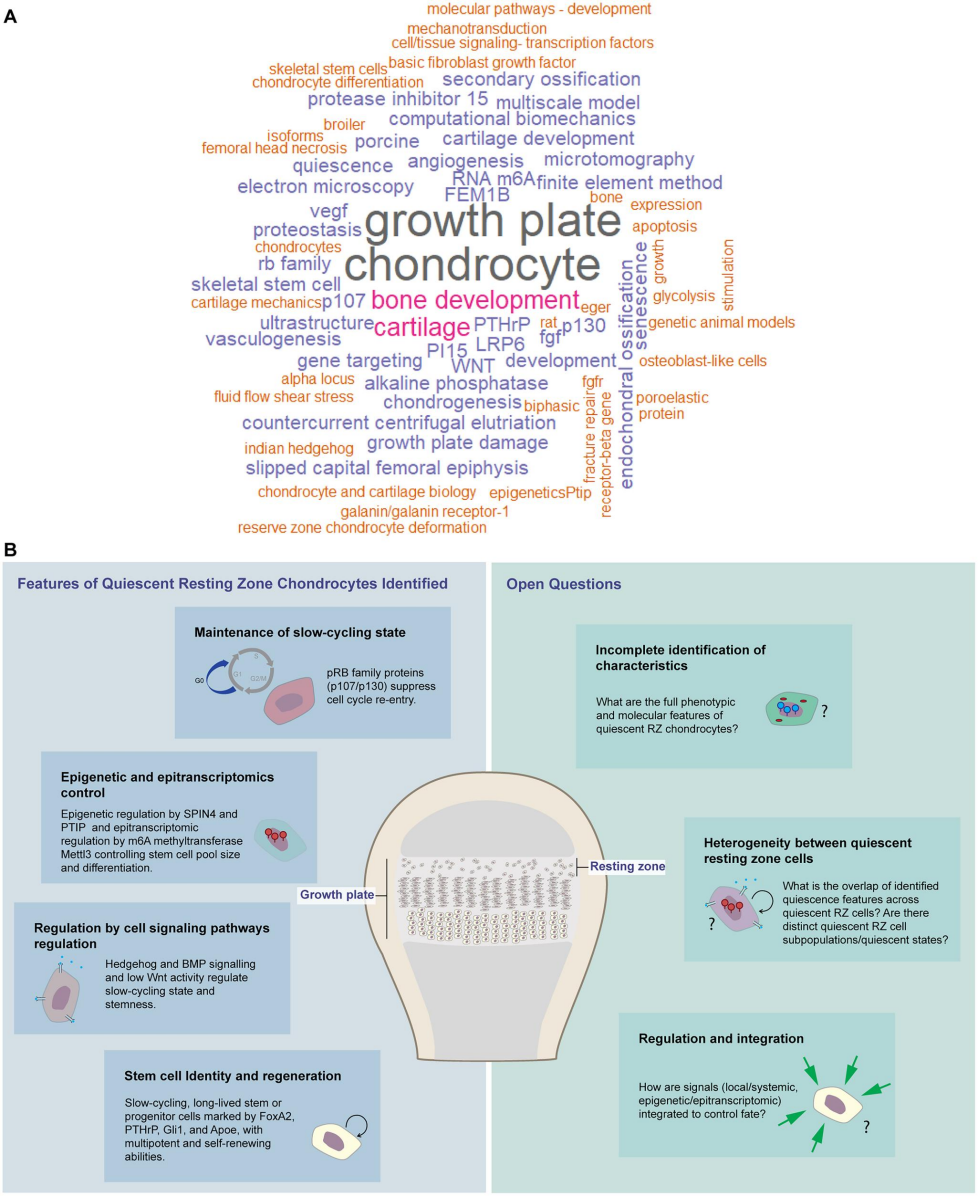
Author keywords distribution and features of quiescent RZ chondrocytes and open questions in the field. (A) Visualizing the keywords chosen by authors from Embase and Web of Science databases. The size of each word corresponds to its frequency, providing a visual representation of the main themes and concepts explored in this systematic review. See also Figures S3 and S4. (B) The schematic summarizes the current understanding of quiescent RZ chondrocytes within the growth plate (central figure), highlighting identified features (left panels) and emerging questions (right panels), underscoring both progress in RZ chondrocyte research and the need for future studies to resolve outstanding challenges.

To explore research themes within the literature, we generated a word cloud from author-provided keywords in Embase and Web of Science (Figure 2A). The most frequent terms were “chondrocyte” and “growth plate” which reflected the primary focus on skeletal biology. In contrast, terms related to the “resting zone” (including “germinal” or “reserve zone”) and “skeletal stem cells” were largely missing. Only one study contained “reserve zone chondrocyte deformation”,^65^ and “quiescence” appeared in a single study.^82^ Of the 25 studies analyzed, 15 included author keywords.^53^^,^^55–58^^,^^60^^,^^63–65^^,^^68^^,^^70^^,^^73^^,^^75^^,^^82^^,^^83^ While these keywords help illustrate each study’s scope, they lack standardization, leading to inconsistencies in terminology (eg, “resting zone” vs “reserve zone”) and making comprehensive literature searches more challenging.^86^^,^^87^ To broaden this assessment, we also examined Web of Science Keywords Plus and Embase Medical Index Terms, found in 14 and 21 studies, respectively (Figures S3-4). Both systems aid literature organization and discoverability: Keywords Plus identifies related terms from cited references,^88^^,^^89^ while Embase Index Terms offer greater precision through controlled vocabulary.^90^ Notably, only one study^52^ was tagged with “resting zone” in Keywords Plus, and no standardized keyword was available in PubMed or Embase, highlighting the limited research focus on RZ chondrocyte quiescence.

#### Research findings

The results from Table 1 revealed that cellular quiescence in the RZ was investigated in 12 of the identified studies; we extracted the research findings relating to quiescent RZ cells from these studies (Table 2). The results revealed several key features of quiescent RZ cells that could be categorized as relating to stem cell identity, lineage potency, regeneration capacity, the mechanical regulation of the slow-cycling state, the regulation of cell cycle progression in RZ by cell signaling pathways, epigenetic regulation of slow-cycling state and differentiation potential, metabolic status, and cell size. We made detailed extractions of these experiments and results (Note S4), and in the following paragraphs isolated important elements for synthesis.

**Table 2.** Summary of experimental features from studies investigating cellular quiescence in the RZ.

Study	Was quiescence in RZ studied?	Experimental approaches	Species	Age	Methods used to directly study quiescence	Level of evidence
**53**	Yes	In vivo	Mouse	E16-19	Histological and BrdU incorporation in the center of the epiphyseal cartilage of global *p107* and *p130* genetically ablated mice.	D
**49**	Yes	In vivo	Mouse	P11, P15, P27	Conditional gene knockout in mice, histological analysis	D
**3**	Yes	In vivo	Mouse	P9, P12, P21, P36, P96	Genetic pulse-chase approach, RNA-seq	D
**50**	Yes	In vivo and use of unpassaged primary cells	Mouse	up to 1 year	Lineage tracing, injury response analysis	D
**67**	Yes	In vivo	Mouse	P14	Mouse model with *Spin4* ablation	C-D
**69**	Yes	In vivo	Mouse	P21, P36, P42, P56	Mouse model with Hedgehog signaling activation	D
**70**	Yes	In vivo	Mouse	P20	Molecular genetics, spatial transcriptomics	D
**51**	Yes	In vivo and use of unpassaged primary cells	Mouse	P15, P23, P28	Genetic lineage tracing, mouse knockout models	D
**75**	Yes	In vivo	Mouse	P3, P14, P17, 12-week	RNA-seq, ChIP-seq, Genetic lineage tracing, mouse knockout model, BrdU labeling	D
**80**	Yes	In vivo	Mouse	P11, P23, P26, P28, P36, 9-week, 1-year	Mouse knockin model, BrdU labeling	D
**82**	Yes	In vivo and use of unpassaged primary cells	Mouse	P3, P14, 1-month, 3-month,	SLIM-seq, mouse knockout model, mouse knockdown model, BrdU labeling	D
**83**	Yes	In silico	Mouse	Day 2—p9 Day16—p28	Continuum-based particle model	D

E = embryonic day; P = postnatal day. Level of evidence was graded according to published guidelines.^91^

While RZ chondrocytes are relatively quiescent as a population, individual cells in the RZ of rodents frequently undergo cell division.^51^ The sub-populations of RZ chondrocytes at varied quiescence states have not been characterized in articles that appeared in our literature search. Although numerous studies, including those identified here, have revealed that RZ cells have heterogenous marker expression,^49–51^^,^^53^^,^^67^^,^^69^^,^^70^^,^^75^^,^^80^^,^^82^ neither scRNAseq analyses of wild-type mice at postnatal day 28 (p28),^80^ p2—12 weeks of age^75^ nor LRCs^3^ have thus far not revealed whether the RZ contains sub-populations of quiescent cells.

The hierarchy of RZ stem cells is also yet to be fully established; whereas several of the studies identified specific markers, such as FoxA2,^50^ Gli1,^51^ and Apoe,^80^ their expression in relation to quiescence status of the cells is not clear. Fate mapping in mice showed that FoxA2+ cells can persist as individual cells for prolonged lengths of time, indicating a functional quiescent behavior, but this population are also able to form new chondrocyte columns, and can give rise to Parathyroid Hormone-Related Protein (PTHrP)+ cells,^50^ indicating a hierarchical relationship among stem/progenitor pools.^2^

Using a conditional genetic ablation approach (FoxA2CreERT2/+; DTA fl/fl mice) with tamoxifen-induced deletion of FoxA2+ cells, Muruganandan and colleagues demonstrated that loss of these cells at early postnatal stages (p4-p18) significantly impaired cartilage regeneration following growth plate injury (Salter-Harris type I fracture), as measured by reduced tissue repair and decreased proliferation and migration of FoxA2+ cells.^50^ This result suggests that FoxA2+ RZ chondrocytes can be activated to exit quiescence and contribute to bone elongation under certain conditions.

Since cellular quiescence is primarily defined by a slow rate of cell division, the findings that the mechanical regulation of RZ cell cycle progression relies on p107 and p130 remain highly relevant. Unsurprisingly, many of the studies utilize changes in cell division as a read out for altered quiescence level. This includes six studies that provide insights into how hedgehog signaling regulates the slow-cycling state; for example that both Gsα and Gq/11α signaling downstream of the PTH/PTHrP receptor are required to sustain this non-dividing state,^49^ that the adhesion G-protein coupled receptor Adgrg6 is essential for maintaining the PTHrP+ slow-cycling RZ via hedgehog signaling,^70^ and the increased clonal sizes of PTHrP-labeled chondrocytes upon tamoxifen-inducible ablation of Ptch1.^69^ Further use of inducible Cre-loxP based systems to impair Bone Morphogenic Protein (BMP) signaling in Gli1-positive cells and activation of Wnt signaling in PTHrP-positive cells indicated their importance to maintaining RZ cell quiescence. More recent studies have identified modifiers leading to changes in quiescence status based on cell division and differentiation capacity beyond cell signaling pathways, both at the epigenetic^67^^,^^75^ and epitranscriptomic levels.^82^

Various stimuli activate distinct signaling pathways to induce cellular quiescence. For example, in vitro, nutrient deprivation triggers AMPK/mTOR pathway inhibition,^92^^,^^93^ contact inhibition activates Hippo/YAP signaling,^94^ and growth factor withdrawal upregulates Cyclin-Dependent Kinase (CDK) inhibitors like p21 and p27.^7^ In vivo, however, the process may be more complex, with cells entering or exiting quiescence in response to dynamic microenvironmental cues, including growth factors, mechanical signals, and metabolic conditions.^95^ In the growth plate, our literature search revealed that BMP signaling through BMPR1A appears to maintain quiescence whereas stimulation of hedgehog and Wnt signaling pathways causes cells to exit their quiescent states. This is not the entire story, however, as articles not appearing in our literature search also address the maintenance of the RZ population, and have relevance to cellular quiescence, for example, through estrogen,^96^ growth hormone,^97^ and mTORC1 signaling.^4^ While important recent studies have revealed that epigenetic regulation is important to maintain RZ quiescence,^67^^,^^75^ they highlight the need for further investigations to reveal the fine-tuning that epigenetic regulation may have upon RZ quiescence. Whether or not epitranscriptomic regulation beyond N6-methyladenosine (m^6^A) mRNA modifications regulates quiescence of RZ cells also warrants additional research. A relevant knowledge gap that we identified was that the reviewed studies did not directly address the integration of systemic physiological signals with local regulatory networks in the control of the slow-cycling progenitor pool. It is plausible that systemic factors that are vital for bone health and growth, such as nutrition,^98^ hormones,^99^ inflammation,^100^ or mechanical loading,^101^ may interact with local actors, like Wnt, Hedgehog, BMP, PTHrP, and/or epigenetic modulators, to fine-tune the size, and activity of quiescent RZ cells.

## Discussion

Here, we sought to clarify the understanding of RZ chondrocytes as “quiescent” by identifying the use of the prefix “quiescen” in relation to the RZ using systematic review, the results of which led us to several notable findings.

Firstly, despite the perceived importance of cellular quiescence to RZ cells, it was not necessarily clear what authors meant by this term as none of the identified studies explicitly defined the term and less than half included a reference as way of explanation. Analysis of Key Words also led us to understand that cellular quiescence was not a key component of the studies as perceived by the researchers themselves. A limitation of this systematic review is that other papers, relevant to cellular quiescence in RZ, but that do not specifically mention “quiescence,” were not collected by our search strategy; however, had those studies included the prefix “quiescen*” in the keywords, they would have been included. Therefore, the systematic review highlighted both the lack of depth in the understanding of cellular quiescence in RZ and that few studies have specially focused on cellular quiescence in relation to RZ cells.

Research into cellular quiescence in other tissues has developed in recent years so that concepts and features that can be attributed to quiescent cells have greatly advanced. Whereas earlier studies related principally to cell division dynamics,^7^^,^^102^ research in the past 15 years has substantially added to this knowledge to include, for example, mRNA levels and sub-cellular localization,^103^ reliance on glycolysis,^104^ heterochromatin density,^17^ lysosomal properties,^40^ features of nuclear pores.^105^ In line with our expectations, the features of cellular quiescence most associated with RZ cells were their low cell division rates, while “stemness” has been included in several studies in the last decade. We were interested to note that cell size was also used as a description of the RZ cells. Despite this, we found no subsequent studies presenting cell size in RZ cells and were unable to find a citation that established this point. Importantly, features of quiescent cells found in other tissues were not thoroughly described in RZ cells; this includes small cell size, reduced RNA/protein content, suppressed mRNA transcription, low levels of cyclins (A2, B1, E2) and high levels of CDK inhibitors (p21, p27, p57), dense heterochromatin, low mitochondrial and reduced oxidative metabolism, a metabolic reliance on glycolysis rather than oxidative phosphorylation, lysosomal size and number. Hence, in contrast to the well-characterized quiescence features described above, those quiescent RZ chondrocytes are still emerging and thus there is scope for further investigation.

Although the quiescence features of RZ cells are not as well characterized as those of quiescent cells in other tissues, recent progress has revealed significant heterogeneity among RZ chondrocyte populations.^50^^,^^51^^,^^80^ This raises important questions: what is the overlap of (identified and yet to be identified) quiescence features across all RZ cells, and do distinct quiescent subpopulations or states exist? Understanding whether RZ markers, such as FoxA2 or Apoe, play functional roles in sustaining quiescence—and how this integrates with observed heterogeneity—remains an important area for future investigation.

While the identified studies revealed multiple regulators of RZ cell quiescence (summarized in the final paragraph of “Research findings” section), these remain relatively isolated findings; further integration of these signals such as understanding the interaction between downstream signaling pathways, how these signals are integrated via epigenetic/epitranscriptomic alterations, and how cell cycle control is directly regulated by modulating cellular machinery, would greatly expand the understanding of quiescence maintenance and stem cell fate in postnatal skeletal growth.

RZ quiescence may be viewed as an apex in the production of new progenitors within the growth plate and therefore its modulation offers a potentially broad therapeutic target to treat growth disorders. Indeed, genetic and pharmacological perturbation of identified regulators, such as PTHrP,^49^^,^^69^ Wnt,^73^ Hedgehog,^69^^,^^70^ BMP signaling, and epigenetic modifiers like Ptip^75^ or METTL3,^82^ already demonstrates that alterations in quiescence can directly impact RZ stem cell proliferation, clonal output, lineage allocation, and regenerative capacity following injury.^48^ While it may be anticipated that pharmacologically reinforcing quiescence could produce fewer new cells and potentially cause height reduction, in other instances, such as chronic stress, it may preserve the RZ stem cell reservoir. Conversely, releasing RZ cells from quiescence could generate more new chondrocytes but risks stem cell depletion, offering potential short-term growth velocity gains (at the possible cost of early growth plate fusion) or accelerated repair after injury. These possibilities require further research to better understand the identity, regulation and fate of daughter cells, and the need for context-specific modulation to balance progenitor output against stem cell longevity. More subtle modulation of a cell′s quiescence status may also be therapeutically appealing: given it is likely that RZ cells, as with quiescent cells in other tissues,^19^ exist in a continuum, moving along such a continuum could potentially alter behaviors such as ligand secretion, providing a quiescent cell with the means to influence the fate of its neighboring cells without a change in the quiescent cell′s own cell cycle phase. However, although it may seem an attractive target, a better understanding of RZ quiescence is necessary prior to safe therapeutic interventions becoming available. Indeed, RZ perturbation studies,^49^^,^^69^^,^^75^ and conceptual work in other stem cell systems highlight risks such as progenitor exhaustion or aberrant proliferation^15^^,^^19^^,^^20^^,^^30^ if thresholds are exceeded, necessitating further studies to inform safe interventions.

Some limitations of the primary studies should be noted. Of the 12 studies that included experiments, mouse models dominated (92%), limiting human translation. These studies include various approaches to identifying the label retaining RZ population with radiolabeled BrdU,^75^^,^^80^^,^^82^ or to label RZ chondrocytes via, for example, tracing from PTHrP,^49^^,^^50^ Col2a1,^3^ or Foxa2^50^ positive cells, leading to challenges in consolidating results between studies. Considering that none of the primary studies included an explicit definition of RZ chondrocyte “quiescence,” and those which included experiments to study quiescence used different test methods that may be insufficient to reveal the full range of possible quiescence features, these points highlight a poorly understood landscape of RZ quiescence, introducing potential bias and incomplete interpretation within the field.

We evaluated how well the studies enabled us to address our objectives; the overall quality of evidence regarding RZ quiescence features was limited. Of the 25 included primary studies, none explicitly defined “quiescence” (Main objective), with definitions implicitly limited to low cell division rates, small cell size, or stemness (Objective 1). These features were experimentally investigated in only 12 studies (Objective 2), classified as CIViC Level C-D evidence (Table 2),^91^ comprising descriptive histology, genetic lineage tracing, and signaling perturbations. Compared to other stem cell populations discussed previously (eg, HSCs, MuSCs), major gaps remain in characterizing metabolic, epigenetic, and lysosomal quiescence hallmarks (Objective 3). These experimental approaches provide mechanistic support for RZ quiescence regulation, their preclinical nature limits clinical translation; this systematic review underscores the need for functional quiescence assays in human-relevant systems.

Finally, our chronological analysis revealed that ten of the twelve studies examining “quiescence” in the RZ were published after 2021. This recent increase in interest directly follows the findings that epiphyseal stem cells and their niche form in growth plates of postnatal mice.^2^^,^^4^ Stem cell quiescence in various adult tissues is an actively regulated and dynamic state, maintained through complex signaling pathways, metabolic adaptations, and niche interactions. Examples include hematopoietic, muscle, neural, and hair follicle stem cells, where quiescence involves precise control of cell cycle arrest, transcription, and proteostasis.^106–108^ Hence, given the importance of quiescence to stem cell populations throughout the body,^14^ we anticipate continued interest in this research area in the coming years, highlighting a need for further characterization of RZ quiescence features and their control. A summary of identified quiescent features of RZ cells and the questions synthesized by this review are presented in Figure 2B.

## Conclusion

Although cellular quiescence plays a central role in growth plate biology and skeletal development, its definition is often treated implicitly in the literature, with limited discussion of how general and tissue-specific characteristics apply to RZ chondrocytes. Our analysis demonstrated that, compared to other well-studied tissues like neural or hematopoietic stem cell systems, features of cellular quiescence in RZ chondrocytes remain significantly underexplored, with limited molecular and functional characterization. Furthermore, of the characteristics that are described, the extent to which they overlap among the various sub-populations of RZ cells/potential quiescent states is yet to be fully determined. This systematic review can contribute to the overall understanding of skeletal development by thoroughly identifying knowledge gaps and may act to enhance consistency and comparability in future studies of “quiescence” in the RZ chondrocytes.

## Supplementary Material

sxag010_Supplementary_Data

## Data Availability

The data underlying this article will be shared on reasonable request to the corresponding author.
