# Supplementary material for: “Quiescence” in the resting zone of the growth plate: a systematic review
Source: Stem Cells. 2026 Mar 7;44(5):sxag010. doi: 10.1093/stmcls/sxag010 (PMC13110114; doi:10.1093/stmcls/sxag010)
Supplement: sxag010_Supplementary_Data [file sxag010_supplementary_data.zip › Supplementary Information 260225.docx]

**Supplementary Information**

**“Quiescence” in the resting zone of the growth plate: a systematic review.**

Mahtab Avijgan^1 2^, Amal Nazaraliyev^1 2^, Klas Blomgren^1 3^, David Gomez-Cabrero^4^, Phillip T Newton^1 2 #^

Author affiliations:

^1^ Department of Women’s and Children’s Health, Karolinska Institutet; Stockholm, Sweden.

^2^ Astrid Lindgren Children's Hospital, Karolinska University Hospital, Region Stockholm, Stockholm, Sweden.

^3^ Pediatric Oncology, Karolinska University Hospital; Stockholm, Sweden.

^4^ Biological and Environmental Science and Engineering Division, King Abdullah University of Science and Technology KAUST; Thuwal, Saudi Arabia.

Mailing address:

Dept. of Women’s and Children’s Health, Karolinska Institutet, Biomedicum A4, Solnavägen 9, 171 77 Stockholm, Sweden

Author footnotes:

^#^ Corresponding author

Correspondence: [phillip.newton@ki.se](mailto:phillip.newton@ki.se)

Running head: Review of quiescence in resting zone chondrocytes

**Keywords**

Cellular quiescence; Growth plate; Resting zone; Skeletal stem cells; Chondrocyte

**Note 1. The histological organization and functional progression of zones within the growth plate.**

Each growth plate can be histologically divided into different zones based on the functionality of the cells within them. On the epiphyseal side of each growth plate is the resting zone (RZ), which is characterized by slowly dividing cells [109]. The RZ contains stem cells that give rise to transit amplifying chondrocytes in the proliferative zone (PZ) [2]. The PZ contains a stream of flattened cells that undergo several cycles of cell division [103]. Upon leaving the cell cycle, these enter the hypertrophic zone (HZ) as they modulate the extracellular matrix by secreting different cartilage proteins, increase in volume, and mineralize their cartilage matrix [110]. Subsequently the majority of these cells die, and the calcified cartilage remnant is used as a template for bone tissue to be built on [111]. This process continues from birth until we reach our final height, when the growth plate fuses to be replaced entirely by bone tissue [98].

**Note 2. Rationale for excluding passaged cell models in the analysis of RZ quiescence.**

Cellular quiescence can be induced *in vitro* by various stimuli including contact inhibition, reaching Hayflick´s limit and growth factor withdrawal, which act via different signaling pathways [7,112], whereas *in vivo* the situation is likely to be more complex with multiple stimuli holding a cell in quiescence [7]. Since the purpose of this review was to reveal the nature of RZ quiescence *in vivo*, and cell cycle regulation is an integral part of cellular quiescence, we decided that studies involving either cell lines, or primary chondrocytes that had been isolated and passaged would be excluded from this review because of the uncertainty of how closely those cells reflect their *in vivo* counterparts. Please note that relevant studies using un-passaged primary cells or referring to quiescence in un-passaged cells were included. In the case of inclusion, publications were then processed to extract information of a) if the term using the prefix “quiescen” was defined, b) if experiments were conducted to study quiescence, and c) a record of the experimental system was made (Supplementary Figure 1).

**Note 3. Bias-minimization strategies**

Several steps in our design were aimed to minimize bias; two independent reviewers conducted parallel literature searches using validated strategies and a pre-defined validation set of known eligible studies, which ensured a comprehensive identification of relevant records without single-reviewer oversight. Data extraction focused on objective criteria (presence/definitions of "quiescen*" prefix, experimental systems), with chronological/keyword analyses performed using standardized tools (Excel, RStudio) to avoid interpretive bias. Exclusion of passaged cells prioritized in vivo relevance, further reducing methodological heterogeneity.

**Note 4. Below is a comprehensive extraction of research findings.**

Stem cell identity, lineage potency, and regeneration capacity

After identifying FoxA2 expression in resting zone cells by immunofluorescence, the authors genetically traced FoxA2CreERT2 cells in with fluorescent reporter lines [2]; fate mapping showed that FoxA2+ cells can persist as individual cells over time, indicating quiescence, but also that they are able to form new chondrocyte columns, and can give rise to PTHrP+ cells, indicating a hierarchical relationship among stem/progenitor pools [2].

Complementing this, using genetic lineage tracing with Gli1-CreERT2; tdTomato mice, in which tamoxifen was administered at either 1 or 12 months of age, a sub-set of Gli1+ cells were demonstrated to constitute a population of long-lived, slow-cycling chondrogenic progenitors in the growth plate, costal cartilage, and vertebrae. By tracking tdTomato+ descendants over time, Li and colleagues showed that Gli1+ cells robustly contribute to the formation and maintenance of columnar chondrocytes throughout life and exhibit high self-renewal capacity, as confirmed by colony-forming assays and serial passaging. RNA-sequencing of FACS-sorted Gli1+ cells revealed a distinct gene expression profile enriched for skeletal stem/progenitor markers [51].

Kodama and colleagues to map spatial gene expression, identifying 11 distinct cell clusters, including five growth plate chondrocyte populations. By validating marker genes (Acan, Prg4, Epyc, Postn) via FISH, they annotated clusters and discovered Apoe as a pan-marker for RZ chondrocytes using scRNAseq of p28 mouse growth plates with FISH, expressed in >97% of RZ cells with exclusive localization to the histologically defined RZ. To study Apoe+ RZ cells, they generated Apoe:mCherry knock-in mice (genetically engineered to express mCherry under the endogenous Apoe promoter), finding that mCherry+ RZ chondrocytes emerged post-SOC formation (detected at p11, prominent at p28) and declined with aging (peaking at 9 weeks, persisting until 1 year). EdU pulse-chase assays in p14-p36 mice revealed that mCherry+ RZ chondrocytes were slow-cycling (based on EdU retention). Flow cytometry and *in vitro* differentiation of p28 mouse RZ chondrocytes (CD31−CD45−Ter119−CD73+mCherry+) demonstrated their heterogeneity and progenitor potential, including osteogenic/chondrogenic differentiation and colony-forming capacity. Immunofluorescence further showed Apoe expression in human RZ, indicating evolutionary conservation [56].

Collectively, these studies demonstrate that long-living, slow-cycling stem/progenitor cells in the RZ can be labelled with FoxA2, Gli1, and/or Apoe, supporting the notion that the RZ is a critical reservoir of quiescent cells necessary for lifelong skeletal growth, maintenance, and regeneration.

The maintenance of the slow-cycling state

One study elucidated the molecular mechanisms that maintain the slow-cycling state of RZ chondrocytes. The pRB-related proteins p107 and p130 were shown to act redundantly to suppress E2F transcription factor activity, thereby maintaining chondrocyte cell cycle exit in the epiphyseal centers; while single knockouts of either gene were viable, double knockout mice exhibited severe skeletal defects, including shortened limbs and disrupted endochondral bone formation, due to doubled chondrocyte proliferation rates and delayed cell cycle exit, despite preserved terminal differentiation. This underscores the essential role of the pRB family in balancing proliferation and non-proliferation during skeletal development, as their absence results in uncontrolled chondrocyte proliferation and disorganized growth plate architecture [67].

Using scRNAseq Kodama and colleagues identified Ccnd1 (cyclin D1) expression in RZ chondrocytes of mouse growth plate; using FISH, the authors visualized Ccnd1 “at the boundary between unorganized RCs and column-forming proliferating chondrocytes”, which they interpreted as indicative of cells leaving quiescence and entering the cell cycle [56].

Regulation of cell cycle progression in RZ by hedgehog signaling

Six studies provide insights into how hedgehog signaling regulates the slow-cycling, stem-like state of RZ chondrocytes in the growth plate.

Firstly, Chagin and colleagues demonstrated that Gsα signaling in the growth plate is indispensable for maintaining the non-dividing, stem-like state of RZ chondrocytes by using a conditional knockout approach in Col2-CreERt; Gsα fl/fl mice. The authors administered tamoxifen at p3 to induce Gsα ablation specifically in growth plate chondrocytes, and analyzed the resulting effects at multiple postnatal ages, including p8, p12, p16, and up to 3 months. Through BrdU and EdU labeling, Ki67 immunostaining, TUNEL assay, and *in situ* hybridization, they showed that loss of Gsα caused RZ chondrocytes to prematurely exit their slow-cycling state, leading to increased proliferation, accelerated differentiation, depletion of the label-retaining cell pool, and the formation of abnormal, non-proliferative cartilage remnants that persisted for months. Furthermore, by using additional genetic models with selective inactivation of Gsα, Gq/11α, or both, as well as mutant PTH/PTHrP receptor mice, they established that both Gsα and Gq/11α signaling downstream of the PTH/PTHrP receptor are required to sustain this non-dividing state. Loss of either pathway disrupted stem-like chondrocyte maintenance, while combined loss led to apoptosis and growth plate fusion [49].

Bian and colleagues showed that the adhesion G-protein coupled receptor Adgrg6 is essential for maintaining the PTHrP+ slow-cycling RZ pool by supporting SOX9 expression and suppressing Indian Hedgehog (IHH) signaling. To uncover these mechanisms, they used conditional knockout mouse models: Col2a1Cre; Adgrg6 fl/fl for constitutive deletion in osteochondral progenitor cells, and AcanCreERT2; Adgrg6 fl/fl for inducible, postnatal deletion in mature chondrocytes. In the inducible model, tamoxifen was administered after the formation of the RZ, specifically at p6–10, to ablate Adgrg6 in the postnatal growth plate. Through X-ray, microCT, EdU/BrdU labeling, immunohistochemistry, and spatial transcriptomics, they found that loss of Adgrg6 led to premature entry of RZ cells into the cell cycle, early exhaustion of the stem-like pool, and abnormal skeletal growth. These defects were mechanistically linked to increased IHH signaling, which drove expansion of the HZ, while Adgrg6 ablation also resulted in reduced SOX9 and PTHrP expression, confirming the receptor’s critical role in maintaining slow-cycling chondrocyte identity and growth plate homeostasis [71].

Li and colleagues identified that Gli1 was expressed by slow-cycling stem cells and their progenitors in one-month-old mice using Gli1-CreERT2; tdTomato lineage tracing. When Gli1+ cells were labeled with tamoxifen at one month of age and mice were subsequently treated with the hedgehog pathway antagonist vismodegib (GDC-0449), the pharmacological inhibition of IHH signaling disrupted both the proliferation of Gli1+ progenitors and the structural integrity of the growth plate, confirming that IHH signaling is essential for the proliferation and expansion of these chondrogenic progenitors [51].

Orikasa and colleagues further revealed that targeted Hedgehog activation in PTHrP+ RZ chondrocytes disrupts their slow-cycling, non-dividing state, triggering a burst of proliferation, clonal expansion, and increased osteogenic potential. To achieve this, they used a tamoxifen-inducible Pthrp-creER mouse line crossed with Ptch1 fl/fl and tdTomato reporter alleles, allowing for specific and temporal activation of Hedgehog signaling in PTHrP+ RZ chondrocytes. Tamoxifen was administered at p6, and the effects were assessed at multiple time points (p14, p21, and beyond), which led to aberrant clonal expansion, forming distinctive concentric clusters (which the authors named “patched roses”) within the RZ. These cells showed increased entry into the cell cycle, as evidenced by EdU incorporation, and this proliferative response was specific to the RZ. Importantly, the proliferative and hyperplastic state induced by Hedgehog activation was transient: the number and width of clonal columns increased dramatically between p14 and p36, then declined toward p96, with the slow-cycling state then re-established over time [77].

Wang and colleagues used super-low-input m6A sequencing (SLIM-seq) to map m6A RNA modifications across eight skeletal cell populations isolated by FACS from p3 mice using CD45, Ter119, and Tie2 as lineage markers and CD51, Thy1.1/1.2, 6C3, CD105, and CD200 as stem/progenitor markers. They identified 5,327 high-confidence m6A-tagged mRNAs and showed that m6A levels are highest in stem/progenitor populations (SSCs, pre-BCSP, BCSP), with 65% of m6A modifications in mature cells established *de novo*. To test the functional role of m6A, they generated conditional knockout mice (Col2a1-Cre; Mettl3fl/fl) lacking the core m6A methyltransferase Mettl3 in skeletal lineages, which resulted in reduced m6A in SSCs and progenitors, shortened limbs, and disrupted growth plate organization. Phenotypic analyses at multiple time points (p3, p14, 1 and 3 months) using histology, micro-CT, and TRAP staining revealed that Mettl3 deletion led to increased SSC proliferation, expanded growth plate PZ, and progressive bone dysplasia, without affecting osteoclast activity. Flow cytometry, EdU labeling, and gene expression profiling showed that Mettl3KO SSCs exit the non-cycling state and upregulate cell cycle genes, while *in vivo* renal capsule transplantation and *in vitro* osteogenic differentiation assays demonstrated impaired bone formation and reduced osteogenic potential in Mettl3-deficient SSCs. Mechanistically, RNA-seq and SLIM-seq identified upregulation of Fem1b in Mettl3KO SSCs, with m6A mapping, mRNA stability, and functional rescue experiments (Fem1b knockdown) confirming that Mettl3 normally promotes Fem1b mRNA decay. Proteomic (LC-MS, co-IP), ubiquitination, and protein stability assays revealed that upregulated Fem1b in Mettl3KO SSCs targets the transcription factor Gli1 for degradation, impairing SSC function and non-cycling state. Overexpression of Gli1 or activation of Hedgehog signaling (by SAG treatment) restored osteogenic differentiation and partially rescued SSC non-cycling state and growth plate structure in Mettl3KO mice [64]. Thus, through a combination of conditional knockout, cell sorting, transcriptomic, proteomic, and functional assays, they established that Mettl3-dependent m6A modification maintains SSC slow-cycling state and function via a Fem1b-Gli1 axis.

Stoop and colleagues developed a 3D computational model – a continuum-based particle model (CbPM) – to simulate endochondral ossification and the spatial organization of different growth plate zones during bone development. Their model integrated both the mechanical properties of tissue and the biochemical feedback between the PTHrP and Ihh signaling. In their simulations, the primary ossification center (POC) was initiated at day 0, and the SOC began forming at day 2 (in pseudotime). The researchers varied the time it took for the SOC to reach full maturation, testing durations ranging from 0.5 to 16 days. They found that when PTHrP production in the RZ increased rapidly – corresponding to a short SOC maturation period – the growth plate was preserved. In contrast, when PTHrP production increased more slowly – meaning the SOC took a longer time to mature – this delay allowed premature differentiation in the RZ, resulting in early fusion of the growth plate [58]. The authors concluded that a sufficiently fast increase in RZ PTHrP expression following SOC initiation is necessary to prevent growth plate fusion by inhibiting premature differentiation in RZ.

Together, these studies support the notion that hedgehog signaling, acting through multiple molecular components, critically governs the slow-cycling, stem-like state of RZ chondrocytes, ensuring growth plate integrity, stem cell pool preservation, and proper skeletal development [49,51,58,64,71,77].

Regulation of cell cycle progression and differentiation potential in RZ by other signaling pathways

Two of the identified studies provide insights into how BMP and Wnt signaling pathways regulate the slow-cycling, stem-like state of RZ chondrocytes in the growth plate.

Gli1-CreERT2:tdTomato mice were crossed with Rosa26-SAloxP-stop-loxP-DTA mice, and tamoxifen was administered at 1 month postnatally to ablate Gli1+ cells, resulting in severe growth plate disorganization and impaired chondrocyte proliferation and differentiation. To dissect regulatory pathways, the authors generated conditional knockout mice (Gli1-CreERT2; tdTomato; Bmpr1α fl/fl) and deleted Bmpr1α specifically in Gli1+ cells with tamoxifen at 1 month of age, revealing that BMP signaling via BMPR1A is essential for maintaining the slow-cycling, self-renewing state; loss of BMP signaling forced progenitors into transient over-proliferation, followed by rapid exhaustion and structural loss [51].

Hallett and colleagues employed a genetic label-retention system (Col2a1-tTA; TRE-H2B-EGFP) combined with a doxycycline pulse-chase approach to distinguish slow-cycling chondrocytes from their neighbors, and demonstrated that slow-cycling, label-retaining chondrocytes (LRCs) in the RZ are enriched in Wnt inhibitors (including Sfrp5, Wip1 and Dkk2) using flow cytometry and RNA-sequencing. To directly test the role of Wnt signaling, they conditionally activated Wnt/β-catenin signaling in PTHrP+ resting zone chondrocytes by inducing haploinsufficiency of the Apc gene using a Pthrp-creER; Apc fl/+; tdTomato mouse model, administering tamoxifen at p6 and analyzing the effects at multiple time points (p9, p12, p21, p26, and p96). This approach revealed that activation of Wnt signaling impairs the maintenance and differentiation capacity of PTHrP+ chondrocytes based on the number of large clones formed from labelled clones in long-term chase experiments [3].

Epigenetic regulation of slow-cycling state and differentiation potential in RZ

While the importance of epitranscriptomics was outlined above, two of the studies indicate the importance of the epigenetic landscape in maintaining RZ cell quiescence. Firstly, Lui and colleagues uncovered a novel X-linked overgrowth syndrome caused by a hemizygous frameshift variant in SPIN4, identified in a patient with extreme prenatal and postnatal overgrowth, advanced bone age, macrocephaly, and organomegaly, but normal intellectual development [78]. SPIN4 encodes an epigenetic reader that binds specific histone modifications, promotes canonical Wnt/β-catenin signaling, and inhibits cell proliferation; the pathogenic variant abolishes these functions [78]. In *Spin4*-deficient mice, there was a marked increase in the number of RZ progenitor chondrocytes (SFRP5+, CD73+) and EdU+ RZ chondrocytes, without a change in proliferation, indicating accumulation of slow-cycling progenitors rather than heightened proliferation. *Spin4* is most highly expressed in RZ, and mechanistic studies revealed that its loss creates a Wnt-inhibitory environment in the growth plate, favoring maintenance and expansion of the slow-cycling progenitor pool and resulting in generalized overgrowth [78]. Thus, SPIN4 functions as a negative regulator of body growth by binding modified histones, promoting Wnt signaling, and restricting the size of the slow-cycling chondrocyte pool in the RZ; loss-of-function variants disrupt this epigenetic regulation, causing excessive accumulation of slow-cycling progenitors and overgrowth, establishing SPIN4 as the first epigenetic reader gene linked to human overgrowth syndrome [78].

Using H3K27ac ChIP-seq, which identifies active enhancers based on the acetylation of lysine residue 27 on histone 3, Liang and colleagues identified Ptip as the most abundant super-enhancer-associated gene in mouse CD45− Ter119− Tie2− CD51+ Thy1− 6C3− CD105− CD200+ SSCs (isolated from the metaphyses of tibias and femurs from p3 mice from which the bone marrow and periosteum had been surgically dissociated) [63]. Conditional deletion of Ptip in Col2-cre; Ptip fl/fl mice resulted in significantly reduced bone lengths and expanded HZ at p3. Ptip loss altered the relative abundance of the SSCs. Epiphyseal cartilage contained different proportions of cells, indicating altered differentiation potential, a notion supported by studies using passaged primary cells. By p14, expansion of the RZ, PZ, and HZ of the growth plate was observed and by p30 irregular chondrocyte columns, abnormal hypertrophic zone and reduced bone mass were reported. BrdU staining showed Col2-cre; Ptip fl/fl mice had a higher percentage of proliferating cells in the growth plate at p30. Hence, these results indicate that epigenetic regulation of Ptip is an important mediator of RZ cell function.

Metabolic pathways in RZ chondrocytes:

In the experiments described in the section above, Ptip-deficient SSCs isolated from Col2-cre; Ptip fl/fl mice at p3 by Liang and colleagues had elevated expression of glycolytic genes, including Pgk1, versus controls [63]. Although *in vitro* experiments using sub-cultured SSCs transfected with *Pgk1*-shRNA suggested that Pgk1 mediated glycolysis in those cells, *in vivo* application of glycolysis inhibitors to Col2-cre; Ptip fl/fl mice could not rescue bone length deficiencies by p14, although associated phenotypes (including HZ height and bone mass) were alleviated. This suggests that loss of PTIP in chondrocytes may reduce bone length independently of glycolysis. Further research is needed to fully understand specific metabolic conditions in quiescent RZ cells.

RZ cell size:

Although two of the 25 studies [61,62] used small cell size as a description of quiescence (Table 2), we did not find any experiments exploring RZ cell size or volume in the research results.
