# Supplementary material for: “Quiescence” in the resting zone of the growth plate: a systematic review
Source: Stem Cells. 2026 Mar 7;44(5):sxag010. doi: 10.1093/stmcls/sxag010 (PMC13110114; doi:10.1093/stmcls/sxag010)
Supplement: sxag010_Supplementary_Data [file sxag010_supplementary_data.zip › Supplementary Table 1.docx]

**Supplementary Table 1. Search Methodology.**

| 1. Reviewer 1 search terms |
| --- |
| - 1. Free Texts: |
| “Resting zone” |
| “Reserve zone” |
| “Germinal zone” |
| "Resting chondrocyte" |
| "Progenitor chondrocyte" |
| epiphys* |
| “Growth plate” |
| "Growth Plates" |
| "Epiphyseal Plate" |
| "Epiphyseal Plates" |
| "Epiphyseal Cartilage" |
| "Epiphyseal Cartilages" |
| - 1. Standardized Keywords: |
| - - 1. MeSH (PubMed only): |
| "Growth Plate"[mesh] |
| "growth plate/metabolism"[mesh] |
| "growth plate/cytology"[mesh] |
| "chondrocytes/metabolism"[mesh] |
| "chondrocytes/cytology"[mesh] |
| "chondrogenesis/genetics"[mesh] |
| - - 1. Emtree (Embase only): |
| 'epiphysis plate'/exp |
| epiphysis plate'/mj |
| chondrocyte'/exp |
| chondrocyte'/mj |
| 'chondrogenesis'/exp |
| chondrogenesis'/mj |
| - - 1. Author Keywords/Keywords Plus (Web of Science only): |
| "Chondrogenic Progenitors" AK |
| "Chondrogenic Progenitors" KP |
| "Stem Cell Maintenance" AK |
| "Stem Cell Maintenance" KP |
| "CHONDROCYTE PROLIFERATION" AK |
| "CHONDROCYTE PROLIFERATION" KP |
| "GROWTH-PLATE" AK |
| "GROWTH-PLATE" KP |
| "CHONDROGENESIS" AK |
| "CHONDROGENESIS" KP |
| "Skeletal stem cells" AK |
| "Skeletal stem cells" KP |
| "RESTING ZONE" AK |
| "RESTING ZONE" KP |
| 1. Reviewer 2 search terms: |
| - 1. Free Texts: |
| quiesce* |
| epiphys* |
| chondro* |
| "growth plate" |
| cartilage |
| "resting zone" |
| "reserve zone" |
| "germinal zone" |
| 1. Reviewer 1 search strategies: |
| - 1. PubMed |
| (((((("Growth Plate"[mesh]) OR ("growth plate/metabolism"[mesh])) OR ("growth plate/cytology"[mesh])) OR ("chondrocytes/metabolism"[mesh])) OR ("chondrocytes/cytology"[mesh])) OR ("chondrogenesis/genetics"[mesh])) AND (((((("Resting zone") OR ("Reserve zone")) OR ("Germinal zone")) OR ("Resting chondrocyte")) OR ("Progenitor chondrocyte")) AND (((((((epiphys*) OR ("Growth plate")) OR ("Growth Plates")) OR ("Epiphyseal Plate")) OR ("Epiphyseal Plates")) OR ("Epiphyseal Cartilage")) OR ("Epiphyseal Cartilages"))) |
| - 1. Embase |
| ((('resting zone' OR 'reserve zone' OR 'germinal zone' OR 'resting chondrocyte' OR 'progenitor chondrocyte') AND (epiphys* OR 'growth plate' OR 'growth plates' OR 'epiphyseal plate' OR 'epiphyseal plates' OR 'epiphyseal cartilage' OR 'epiphyseal cartilages')) AND ('epiphysis plate'/exp OR 'epiphysis plate'/mj OR 'chondrocyte'/exp OR 'chondrocyte'/mj OR 'chondrogenesis'/exp OR 'chondrogenesis'/mj)) AND 'article'/it |
| - 1. Web of Science search strategy |
| (ALL=(“Resting zone” OR “Reserve zone” OR “Germinal zone” OR "Resting chondrocyte" OR "Progenitor chondrocyte") AND ALL=(epiphys* OR “Growth plate” OR "Growth Plates" OR "Epiphyseal Plate" OR "Epiphyseal Plates" OR "Epiphyseal Cartilage" OR "Epiphyseal Cartilages") AND (ALL=("Chondrogenic Progenitors" AK) OR ALL=("Chondrogenic Progenitors" KP) OR ALL=( ''Stem Cell Maintenance" AK) OR ALL=( "Stem Cell Maintenance" KP) OR ALL=("CHONDROCYTE PROLIFERATION" AK) OR ALL=("CHONDROCYTE PROLIFERATION" KP) OR ALL=("GROWTH-PLATE" AK) OR ALL=("GROWTH-PLATE" KP) OR ALL=("CHONDROGENESIS" AK) OR ALL=("CHONDROGENESIS" KP) OR ALL=("Skeletal stem cells" AK) OR ALL=("Skeletal stem cells" KP) OR ALL=("RESTING ZONE" AK) OR ALL=("RESTING ZONE" AK))) |
| 1. Reviewer 2 search strategies: |
| - 1. quiesce* epiphys* |
| quiesce* chondro* |
| quiesce* "growth plate" |
| quiesce* cartilage |
| quiesce* "resting zone" |
| chondro* "resting zone" |
| chondro* "reserve zone" |
| chondro* "germinal zone" |
