# Supplementary material for: “Quiescence” in the resting zone of the growth plate: a systematic review
Source: Stem Cells. 2026 Mar 7;44(5):sxag010. doi: 10.1093/stmcls/sxag010 (PMC13110114; doi:10.1093/stmcls/sxag010)
Supplement: sxag010_Supplementary_Data [file sxag010_supplementary_data.zip › Supplementary Table 3.docx]

**Supplementary Table 3. Characteristics of excluded studies.**

| **No.** | **Found by** | **Study** | **Reason for exclusion** |
| --- | --- | --- | --- |
| 1 | Both reviewers | Zhang, 2005, Developmental biology | Not referring to quiescence in resting zone cells. |
| 2 | Both reviewers | Chen, 1999, Journal of Clinical Investigation | Not referring to quiescence in resting zone cells. |
| 3 | Both reviewers | Li, 1999, Human Molecular Genetics | Not referring to quiescence in resting zone cells. |
| 4 | Both reviewers | Mundlos, 1994, Developmental dynamics: an official publication of American Association of Anatomists | Not referring to quiescence in resting zone cells. |
| 5 | Both reviewers | Sylvia, 2001, Journal of Cellular Biochemistry | The quiescence referred to passaged resting zone cells. |
| 6 | Reviewer 2 | Fukuo, 1989, Cell Calcium | Not referring to quiescence in resting zone cells. |
| 7 | Reviewer 2 | Takano, 1983, Journal of Biochemistry | Not referring to quiescence in resting zone cells. |
| 8 | Reviewer 2 | Chacko, 1969, Journal of Experimental Science | Not referring to quiescence in resting zone cells. |
| 9 | Reviewer 2 | Angelozzi, 2021, Methods of Molecular Biology | Not an original research article. |
| 10 | Reviewer 2 | Closs, 1990, The Journal of Cell Biology | Not referring to quiescence in resting zone cells. |
| 11 | Reviewer 2 | Lee, 2001, Mechanisms of Development | Not referring to quiescence in resting zone cells. |
| 12 | Reviewer 2 | Kocialkowski, 2001, Anatomy and Embryology | Not referring to quiescence in resting zone cells. |
| 13 | Reviewer 2 | Lee, 2001, Developmental Biology | Not referring to quiescence in resting zone cells. |
| 14 | Reviewer 2 | Falsafi, 2000, Archives of tolaryngology – head & neck surgery | Not referring to quiescence in resting zone cells. |
| 15 | Reviewer 2 | Hiraki, 1986, European Journal of Biochemistry | Not referring to quiescence in resting zone cells. |
| 16 | Reviewer 2 | Forristal, 2014, Molecular and Cellular Biology | Not referring to quiescence in resting zone cells. |
| 17 | Reviewer 1 | Horan, 2009, Journal of Bone and Mineral Research | The quiescence referred to passaged resting zone cells. |
| 18 | Reviewer 1 | Erickson, 1997, Journal of Orthopaedic Research | Not referring to quiescence in growth plate. |
| 19 | Reviewer 1 | Pedrozo, 1999, Journal of Bone and Mineral Research | The quiescence referred to passaged resting zone cells. |
| 20 | Reviewer 1 | Nasatzky, 1999, Endocrine | The quiescence referred to passaged resting zone cells. |
| 21 | Reviewer 1 | Schwartz, 2000, Journal of cellular physiology | The quiescence referred to passaged resting zone cells. |
| 22 | Reviewer 1 | Sylvia, 2000, The Journal of Steroid Biochemistry and Molecular Biology | The quiescence referred to passaged resting zone cells. |
| 23 | Reviewer 1 | Nasatzky, 2000, ENdocrine | The quiescence referred to passaged resting zone cells. |
| 24 | Reviewer 1 | Schwartz, 2002, Endocrinology | The quiescence referred to passaged resting zone cells. |
| 25 | Reviewer 1 | Boyan, 2003, Journal of cellular biochemistry | The quiescence referred to passaged resting zone cells. |
| 26 | Reviewer 1 | Schwartz, 2004, Biochimica et Biophysica Acta | The quiescence referred to passaged resting zone cells. |
| 27 | Reviewer 1 | Raz, 2005, Journal of cellular biochemistry | The quiescence referred to passaged resting zone cells. |
| 28 | Reviewer 1 | West, 2006, Journal of orthopaedic research: official publication of the Orthopaedic Research Society | Not using quiescence term in main text. |
| 29 | Reviewer 1 | Boyan, 2006, Journal of bone and mineral research: the official journal of the American Society for Bone and Mineral Research | The quiescence referred to passaged resting zone cells. |
| 30 | Reviewer 1 | Hurst-Kennedy, 2009, Biochimica et Biophysica Acta | The quiescence referred to passaged resting zone cells. |
| 31 | Reviewer 1 | Schwartz, 1998, Endocrinology | The quiescence referred to passaged resting zone cells. |
| 32 | Reviewer 1 | Kieswetter, 1997, Endocrine | The quiescence referred to passaged resting zone cells. |
| 33 | Reviewer 1 | ElBaradie, 2012, The Journal of Steroid Biochemistry and Molecular Biology | Not referring to quiescence in growth plate. |
| 34 | Reviewer 1 | Schwartz, 1999, Endocrinology | The quiescence referred to passaged resting zone cells. |
| 35 | Reviewer 1 | Schwartz, 1998, Endocrinology | The quiescence referred to passaged resting zone cells. |
